# Quantitative Structure Retention-Relationship Modeling: Towards an Innovative General-Purpose Strategy

**DOI:** 10.3390/molecules28041696

**Published:** 2023-02-10

**Authors:** Priyanka Kumari, Thomas Van Laethem, Philippe Hubert, Marianne Fillet, Pierre-Yves Sacré, Cédric Hubert

**Affiliations:** 1 Department of Pharmacy, Laboratory of Pharmaceutical Analytical Chemistry, University of Liège (ULiege), CIRM, Quartier Hopital (B36 Tower 4), Avenue Hippocrate, 4000 Liège, Belgium; 2 Laboratory for the Analysis of Medicines, University of Liège (ULiege), CIRM, Quartier Hopital (B36 Tower 4), Avenue Hippocrate, 4000 Liège, Belgium

**Keywords:** QSRR, machine learning, stacking, applicability domain, RPLC

## Abstract

Reversed-Phase Liquid Chromatography (RPLC) is a common liquid chromatographic mode used for the control of pharmaceutical compounds during their drug life cycle. Nevertheless, determining the optimal chromatographic conditions that enable this separation is time consuming and requires a lot of lab work. Quantitative Structure Retention Relationship models (QSRR) are helpful for doing this job with minimal time and cost expenditures by predicting retention times of known compounds without performing experiments. In the current work, several QSRR models were built and compared for their adequacy in predicting the retention times. The regression models were based on a combination of linear and non-linear algorithms such as Multiple Linear Regression, Support Vector Regression, Least Absolute Shrinkage and Selection Operator, Random Forest, and Gradient Boosted Regression. Models were built for five pH conditions, i.e., at pH 2.7, 3.5, 6.5, and 8.0. In the end, the model predictions were combined using stacking and the performances of all models were compared. The k-nearest neighbor-based application domain filter was established to assess the reliability of the prediction for further compound prioritization. Altogether, this study can be insightful for analytical chemists working with RPLC to begin with the computational prediction modeling such as QSRR to predict the separation of small molecules.

## 1. Introduction

Liquid chromatography (LC) is widely used in the context of identification and assay of analytes present in a mixture. Several modes such as normal phase liquid chromatography (NPLC), reversed-phase liquid chromatography (RPLC), or hydrophilic interaction liquid chromatography (HILIC) are available. All these modes are based on the same principle, where analytes are present in a liquid mobile phase and are passed through a column containing solid stationary phase under high pressure. The retention time (t_R_) observed is the time taken by the analyte to travel across the column, and is dependent on the difference in the interaction of the analyte with mobile and stationary phases at varied conditions. Several experimental parameters may influence these interactions, leading to a separation of the compounds. Among these, the composition of the mobile phase (i.e., pH, organic modifier, gradient elution) and the stationary phases must be selected. Given the multiple possibilities, finding an optimal condition for such separation is generally performed on a trial-and-error basis and largely depends on the researcher’s prior knowledge. This, in turn, becomes time- and resource-consuming and represents a significant bottleneck of LC analysis in many domains [1]. Quantitative Structure-Retention Relationships (QSRRs) modeling was proposed as an alternative solution to optimize the method development phase [2,3].

QSRR models are computational models that establish a statistically significant relationship between a chromatographic retention parameter and molecular descriptors, which are numerical quantities carrying physico-chemical information of the molecules [4]. Such prediction models could be applied to any type of separation analysis irrespective of the chromatographic techniques or even the modes of a particular technique. Hence, its application range covers many interesting systems such as TLC [4], GC [5], IC [6], RP-LC [7,8], and HILIC chromatography modes [9].

QSRR model development not only enlarges the range of applications, but also increases the understanding of the separation mechanisms. There are several ways of QSRR modeling including the models based on mechanistic equations [10] or based on machine learning methods. The latter are quite popular because of their efficiency and the availability of multiple algorithms. The support vector (SVR) and Partial Least Square (PLS) models are the most popular options [1,11,12,13,14,15,16], but other types of regression algorithms such as Gradient Boosting Regression (GBR), Random Forest, Neural networks, etc. have been successfully applied [17,18,19,20].

Most of the recent machine learning algorithms can be severely limited in accuracy and applicability by the size and nature of the dataset, number, type of descriptors, etc. However, the LC datasets are generally small because of the time and resources needed to build it. Therefore, most modeling strategies imply a feature selection step to avoid overfitting and ensure sparsity of the models since sparse models are generally more robust. Hence, multiple strategies of descriptor selections have been used and shown to have performing differently on different datasets. A feature selection comparison study proposed by Goodarzi et al. showed that models built on descriptors selected by ant colony optimization algorithm coupled with SVR regression could be an excellent alternative for retention prediction modeling [12]. Zuvela–Petar et al. used a PLS regression model built on molecular descriptors selected by a genetic algorithm (GA), particle swarm optimization (PSO), artificial bee colony (ABC), firefly algorithm (FA), and flower pollination algorithm (FPA) [13], whereas Krmar–Jovana et al. compared a combination of linear (MLR) and nonlinear models (SVM) based on a preselected feature set [21]. Pastewska–Monika, et al. and Ulenberg–Szymon, et al. used genetic algorithm coupled with MLR (Multiple Linear Regression) for [22,23]. At the same time, there are models which are based on Bayesian approach that involve using prior knowledge, represented as probability distributions, to make predictions of retention time of a molecule. The prior knowledge is combined with experimental data to produce a posterior probability distribution, which provides a prediction of the retention time. The choice of mechanistic descriptors and the form of the prior distributions can have a significant impact on the accuracy of the predictions made by the model [24,25,26]. Since QSRR models are computational models, prediction discrepancies are frequent because of overfitting which, in turn, questions the reliability of their practical use on new untested chemical compounds. Therefore, it is a good practice to review the model’s validity as per Organization for Economic Co-operation and Development [27]. Although few research studies have checked applicability domain [18,28,29,30], it is still very rare that all QSRR models are accompanied with such validations.

When looking at the literature, the proper well-structured strategy to get started with the structure-derived retention modeling, i.e., the choice of descriptor set, and the selection of a specific regression algorithm are not clearly defined yet. Most studies are based on the researcher’s previous experience or the most cited methods in the literature pool. Hence, a comprehensive generalized overview of the practical strategy when there is a limited dataset, which is the most frequent scenario for such separation studies, would benefit the field of analytical chemistry. Consequently, we propose a strategy that might be used in a variety of cases because of its conception (use of linear, nonlinear algorithms, use of diverse feature selection tools, and applicability domain of the use of selected model). Looking at the current time where deep learning approaches are dominating the ML space, applying them on small dataset is not feasible. Hence, this approach is versatile and useful even on small datasets.

## 2. Results and Discussion

**Figure 1:**
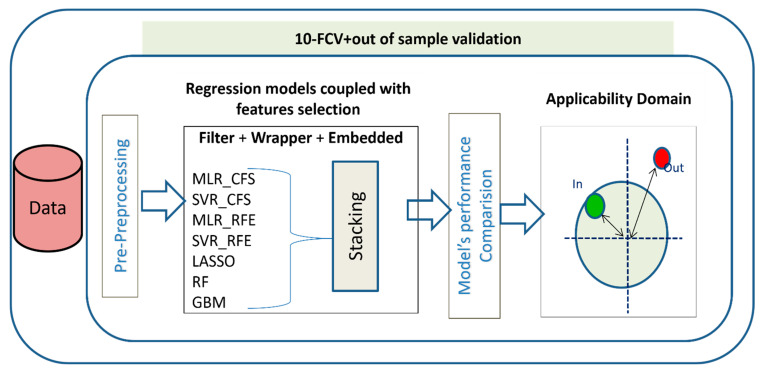
Workflow describing the steps of QSRR Modeling.

In this study a simple, clear, and well-defined strategy (Figure 1) for QSRR modeling is proposed, which can be referred to use when the new test molecule structures are known. Seven diverse machine learning algorithms coupled with three methods of feature selections were evaluated for their retention-time prediction abilities. The regression algorithms and feature selections were chosen based on the fundamental difference in their working mechanism so that the strategy could give a holistic view of the performances of a variety of methods suitable for such predictions. The selected regression algorithms were a combination of linear and nonlinear methods based on single modeling and ensembles, too. Ensemble models were a combination of methods that take advantage of bagging or boosting. The molecular descriptor dataset used for all regression models was varied according to the method of feature selections applied on the dataset. Since linear regression modeling could not handle multicollinearity issue hence, they were coupled with feature selection, before proceeding to regression prediction. These comparative methods provide insights into the applicability of varied models with feature sets for users as to when there will be insufficient or complete lack of domain knowledge, or when there will be a need to support expert knowledge to achieve higher prediction performances with a given set of descriptors.

Preprocessing and feature selection led us to have three types of datasets at each pH: [1] data where features were selected using filter method (e.g., CFS); [2] data where features were selected using wrapper method (e.g., RFE); and [3] data with all features remaining after preprocessing. All datasets were used for regression modeling, and their predictive performances were compared in 10-fold cv and on the external test set.

### 2.1. Diversity of the Dataset

It is expected that the more diverse the dataset, the better the trained models and their generalization performance on the new test set. The diversity of the dataset was checked based on molecular weights and their chemical taxonomy. The molecular weight of the compounds varied from 46 to 456 g/moL. ClassyFire3 [31] was used to obtain a chemical taxonomy of molecules in the dataset using their smile structure (Supplementary File S3). The majority of molecules were classified into eight ClassyFire’s groups on the level of superclass, namely the following: benzenoids (40.0%) organoheterocyclic compounds (29%), organic acids and derivatives (17%), homogenous non-metal compounds (5%), nucleosides, nucleotides and analogues (4%), organic oxygen compounds (2%), phenylpropanoids and polyketides (2%), and other compounds (1%) such as lipids and lipid molecules.

### 2.2. Comparison of Feature Selection Methods

Our data were high-dimensional QSRR datasets, i.e., less data points than number of features. Hence, it was a prerequisite to apply a dimensionality reduction algorithm to make the models computationally less expensive and to improve their prediction performances. In this study, there were three feature selection methods used that were coupled with regression prediction.

(1) Filter methods: In this method, variables were chosen regardless of the model building; hence, these are robust and effective in terms of overfitting and computation time, respectively. These methods work by estimating a relevance score based on a user-defined threshold to select the best-scoring features such as the correlation with the predictive dependent variable [32]. (2) In wrapper method, which is comparatively computationally expensive and prone to overfitting, the variable exists as a wrapper around the predictive model algorithms and uses the same model to select the best features based on some performance measures for example RMSE in this study [32]. (3) The embedded method is a mix of both filter and wrapper methods. Here, the feature selection process is embedded in the learning or the model-building phase and is performed with some penalty on unfavorable features. In other words, these algorithms have an intrinsic strategy of feature selection and overfitting prevention [33,34,35].

In this study, all three categories of feature selection methods were analyzed for their performances in accordance with their use in regression models. From Table 1, Table 2, Table 3, Table 4 and Table 5, the algorithm with feature selections embedded (RF and GBR) and wrapper method RFE performed comparatively better at all pHs. It is also interesting to note that the filter (CFS) and wrapper (RFE) methods, when coupled with non-linear regression methods, perform better than when coupled with linear methods. This could be understood in terms of multicollinearity in the dataset with features. Multicollinearity creates model instability. Better performance of embedded feature selection method could be justified by two arguments: Firstly, they consider the interaction between features giving much closer and detailed information about the data pattern. Secondly, there is no issue of multicollinearity since they apply penalties on correlated features.

### 2.3. Important Features

Every microspecies of molecules exists in dynamic equilibrium during the separation process, and their retention times varies with changing pH. Therefore, the weighted average of their features is expected to give a more informative and descriptive feature set. Good accurate QSRR models at multiple pHs can give us information about the most relevant descriptors for the retention times prediction [36]. Better prediction performance of nonlinear models over linear models inferring those nonlinear patterns of molecular descriptors predicts retention time relatively well. The following steps were followed to make the maximum inference about the selected features: All the features selected using the filter method and wrapper method and the top 20 features used by prediction models (embedded feature selections) were compared. Mutually inclusive features from all the models were selected as the most essential and representative features for retention time predictions. These selected features are listed in Table S4 (Supplementary File S4). LogD, MolLogP and PEOE_VSA6 are the most selected features by all the models at every pH. Apart from these, there were other features such as NHOHCount and VSA_Estate that were also among the selected features. The study has been performed in reverse-phase liquid chromatography, where the difference in the lipophilicity of the compound, is the main factor affecting the retention of the molecules. Hence, the selection of descriptors related to lipophilicity exemplifies better feature selections. LogD and MolLogP are the pH-dependent distribution coefficients and octanol-water partition coefficients, respectively, for every microspecies of a molecule, i.e., both neutral and ionized. PEOE_VSA, which represents the partial atomic charge of the molecule, ranges from 1 to 14 based on the partial charge distribution. In PEOE_VSA parameter, PEOE denotes Partial Equalization of Orbital Electronegativities, which is a charge calculation method, and VSA signifies Van der Waals Surface Area. It is interesting to note that out of 14, it is PEOE_VSA6 which denotes Van der Waals Surface Area having the atomic partial charge in the range of −0.10 to −0.05, which was selected maximally [37]. “NHOHCount” gives the molecule’s NHs and Ohs count, whereas “polarizability” is a measure of electric dipole or electronic charge dispersion in response to an external electric field. These descriptors can, in principle, distinguish between slight differences in a local region of two globally similar molecules. The use of such information, as given by logD, LogP and the PEOE_VSA descriptors, seems necessary to construct a robust and accurate in silico model from structural information of test compounds. These descriptors are a parameterized representation of the hydrophobicity displayed in all modes of RPLC for the separation of varied kinds of analytes.

### 2.4. Predictive Performance of the Different Algorithms on All Datasets

**Figure 2:**
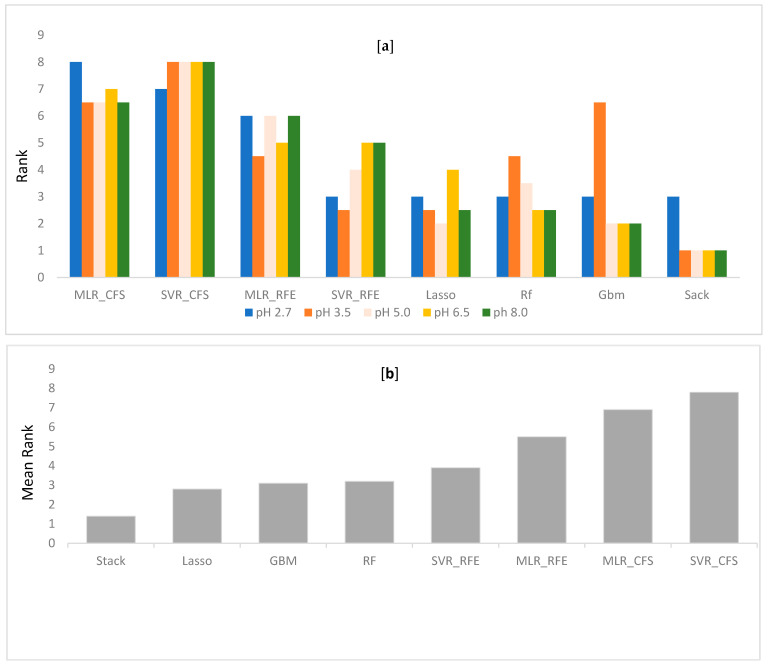
(**a**): Rank of every algorithm based on RMSE along the dataset. (**b**): The mean rank over all datasets when the performance is sorted on RMSE.

**Figure 3:**
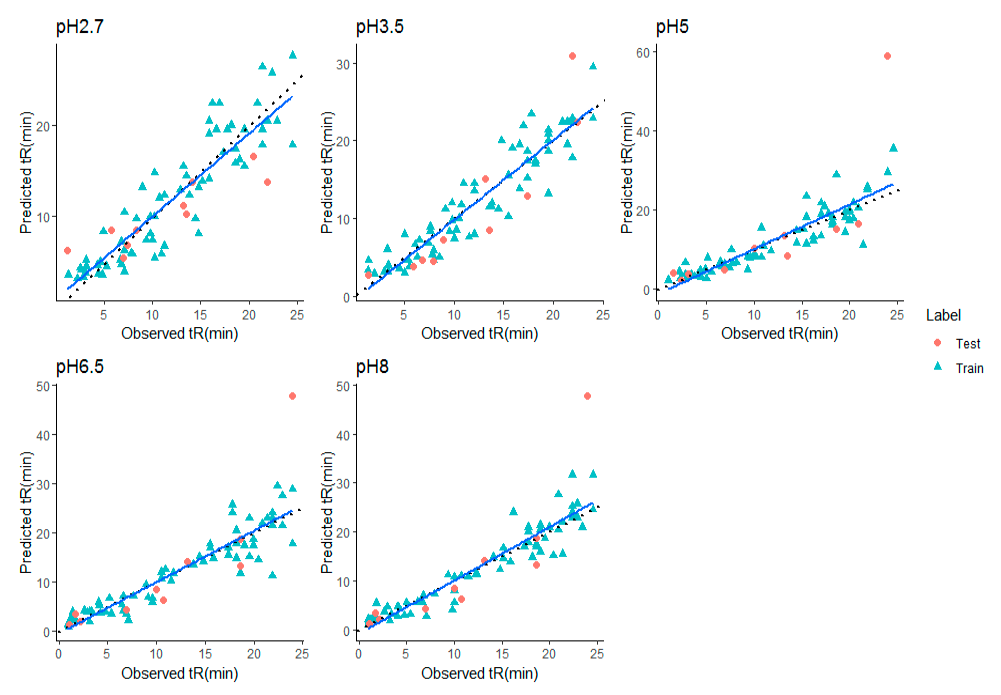
Predicted vs. experimental retention times (in min.) for stacking model at all pHs (Blue line—Fit line, Black dashed line—identity line).

**Figure 4:**
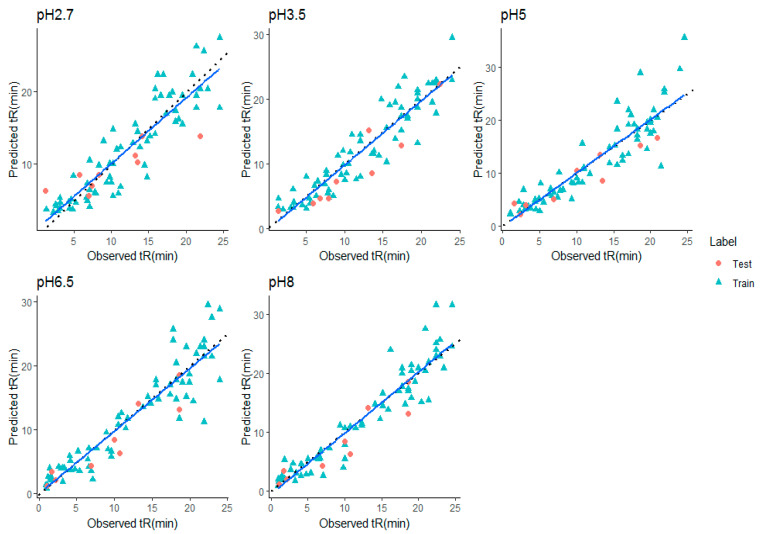
Predicted vs. experimental retention times (in min.) for stacking model at all pHs (after removing Miconazole). (Blue line—Fit, Black dashed line—identity line).

Performance differences between the different QSRR models were evaluated in terms of RMSE and R^2^ on all five datasets. For each dataset, all compounds are used in a nested 10 CV approach to assess the generalization performance. To validate the model, a separate test set of 10 molecules was used. Every model performance on test set was compared at each condition and reported. Grid search method was used in tuning parameters. Tuned parameters for each model at every pH is listed in Supplementary File S10. The detailed CV results for RMSE and R^2^ for each dataset are shown in Table 1, Table 2, Table 3, Table 4 and Table 5, respectively. Mean rank over all datasets (all pH) when the performance was sorted on RMSE, was calculated to find the best suitable model for retention time prediction. From the Figure 2, it is evident that stacking is the best algorithm and hence, can be used for retention time prediction for small molecules in RPLC setup. Linear models such as MLR (CFS, RFE) and LASSO are not performing very well. Figure 2 shows how stacking reduces the RMSE of models over other single models. Note that the ensemble methods like RF and GBM performed comparatively better than single models at lower pH, i.e., at pH 2.7 and 3.5, emphasizing the fact that ensembling is a better way to fit nonlinear relations in a model. The SVR (nonlinear RBF kernel + RFE) model followed after them, performing well for datasets at extreme pH conditions, i.e., at 2.7, 6.5, and 8.0. Stacking performance was comparatively similar to GBM, RF, and SVR_RFE at pH 2.7. Apart from one pH, this algorithm performed consistently well throughout the given pH range. The minimum error of prediction was as less as 0.02. The highest prediction error was observed at pH 2.7. These observations support the fact that except on a few circumstances, out of all algorithms, stacking is most likely to show the better generalization performance. More explanatory discussion about the performance of feature selection coupled with regression models can be provided using observed versus prediction score plots. The closer the fitted line is to the identity line, the better the model. The predicted values and their corresponding experimental retention times for stacking model at all pHs are plotted in Figure 3, and the rest of the models are plotted in Supplementary File S5–S9. Residuals, i.e., the difference between predicted and experimental values for the stacking model, were plotted at all pHs to obtain a closer look at the predictions (Figure 4). The residuals distributions for all dataset validated the superiority of stacking model. Note that, to the authors knowledge, the stacking algorithm has never been applied before for retention time prediction in RPLC.

### 2.5. Applicability Domain Check

It is impossible to anticipate the whole universe of compounds when building a single QSRR model. Hence, there is a need to define the model limitations with respect to its structural domain and response space, which can further be used to evaluate the ambiguity in the prediction of a given molecule relying on the structural similarity of molecules used in the development of the QSRR model. This structural boundary to determine the subspace of chemical structures for reliable property prediction is defined as the applicability domain, which is also the third OECD principle [38]. The query chemicals falling under the defined boundaries of the model are considered within the applicability domain; hence, their predictions will be considered reliable. The predictions of the other molecules which are outside the applicability domain will not be trusted. In cases like this study, where several QSRR models have been built for retention prediction of small molecules, the knowledge of applicability domain helps to compare the reliability of prediction by each QSRR model.

A KNN-fix method (section—applicability domain in material and methods) at a distance of 95% confidence interval was used to define the applicability domain of the QSRR model concerning its structural domain and response space. It is observed that stacking outperformed the rest of the single models; hence, the study of this section was focused on the stacking models only. The error of predictions of all QSRR models for each compound were compared with the distances among features (all features) calculated using the KNN-fix method [39]. It can be seen in Table 6, Figure 3, and Supplementary File S11 that the error of prediction at all pHs was bad for the compound miconazole, which turned out to be out of the applicability domain since its calculated distance was higher than the threshold at every pH.

**Figure 5:**
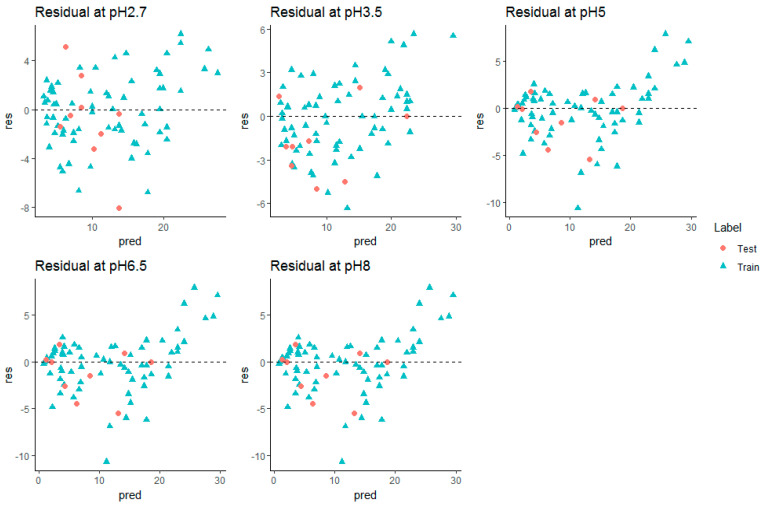
Residual plots (in min.) for stacking model at all pH (without miconazole).

The prediction performance and hence, the regression line and residual plot, was better when plotted (Figure 4 and Figure 5) after removing miconazole from the external test set. Hence, it can be inferred that the retention time prediction of miconazole or any new test compound similar to this cannot be considered reliable. The calculated threshold could serve as a very good measure for filtering new test compounds for retention prediction. Detailed analysis of such behavior of miconazole was beyond the scope of this study.

## 3. Materials and Methods

### 3.1. Dataset Collection

The dataset used in this study was built in-house [40] and consists of retention time observed for 98 small pharmaceutical compounds reported in minutes. The list of small molecules in the dataset came from [41,42,43]. The compounds were tested for their druglikeness (following Lipinski’s rule) using SwissADME tool [44], and more than 90% of the compounds followed all rules of Lipinski’s representing the usefulness of the trained model for other drug-like molecules too. Moreover, the compound selection was performed as such that apart from RPLC they could be relevant for other chromatographic modes such as ionic (IC) and hydrophilic interaction (HILIC). Hence, the strategies developed for one mode can be expandable on another. The data were acquired in RPLC mode using a Waters XSelect HSS T3 (100 × 2.1 mm, 3.5 μm) column at 25 °C, with flow rate 0.3 mL/min at five different pH conditions—2.7, 3.5, 5.0, 6.5, and 8.0—with a gradient elution of 0–95% of methanol in 20 min time.

### 3.2. Molecular Descriptors and Their Calculation

Molecular descriptors play an important role in achieving accurate retention prediction. They form the firm basis for any QSRR model. For regression models, a set of 1D and 2D descriptors covering physical, chemical, and structural properties were calculated for every molecule in the dataset using their SMILE structure taken from PubChem database [45]. The molecular descriptors in this article are calculated taking the ionization state of the compound at the pH of interest into account with the weighted average, where the weights are the percentage of distribution of the microspecies at the considered pH. (described with example in Supplementary File S1). The ionization states were obtained from Chemaxon software (accessed on 4 January)) and the descriptor values were calculated using RdKit library version 2021.09.5. An additional descriptor, logD, was added in the final molecular descriptor set. The value of this descriptor was calculated by Chemaxon at the value of the pH of interest. Thus, a total of 239 molecular descriptors were computed for each molecule at each pH condition (names of descriptors are mentioned in Supplementary File S6).

### 3.3. Data Cleaning and Preprocessing

There are five datasets (varying with pH-2.7, 3.5, 5.0, 6.5, 8.0) used in this study. Each dataset consists of 97 rows and 239 columns initially. All compounds with retention times below 2 min at all pH were removed. Zero-variance descriptors were also removed. Filtered feature names are mentioned in Table S2. Our dataset had features with values of different ranges; hence, the final dataset was standardized before QSRR modeling. The first step involved mean centering, and in the second step data values were divided to standard deviation making the variance of variable to 1. The final dataset had 67 compounds for modeling and 10 compounds in external test set at each pH.

### 3.4. QSRR Modeling with Feature Selection

The choice of regression techniques for correlating structural descriptors with the analyte’s experimental retention time plays crucial role in constructing best and efficient QSRR models. There are no best algorithms defined for such retention predictions. One type of algorithm can work better for one problem, but fail to achieve the same level of accuracy for another. The performance of such regression models depends also on quality of the dataset. Out of many available descriptors, there is a high chance of some redundant, noisy, or irrelevant features in the starting dataset that can create problems in retention prediction: the curse of dimensionality, overfitting problems, high training time for model construction, and poor generalization ability of built models are among a few of these problems [46]. Therefore, a more systematic strategy adapted for QSRR methods is required to determine the possible preliminary, intermediate, and final steps to achieve the absolute accuracy of the best selected QSRR models.

Consequently, a well-organized strategy is proposed here (Figure 1).

Five machine learning algorithms were used—Multiple Linear regression (MLR), Least Absolute Shrinkage and Selection Operator (LASSO), Support Vector Regression (SVR), Random Forest (RF) and Gradient Boosting Regression (GBR). These algorithms were coupled with three feature selection methods: (i) filter (correlation-based filter), (ii) wrapper (Recursive Feature Selection methods, RFE), and (iii) embedded method were compared for their prediction abilities using small molecule datasets.

### 3.5. Combining Multiple Predictions Using Stacking

At the end of the analysis multiple predictions from individual models (Section 3.4) were combined using stacking algorithm. Model stacking is an ensemble method that uses a meta learner to club the predictions from single learners and then combines them to obtain the final predictions [47,48]. Two-level model architecture was used to build a stacking regressor with a hypothesis that, combining individual model’s predictions, would increase the prediction performance. At level 1, all base learners are built and optimized to obtain the best individual predictions. At level 2, the meta learner combines the predictions coming from level-1 models. Predictions made on external test data at level 1 were used to train the meta model. The simplest and most widely used algorithm (MLR) was chosen as meta regressor.

All models were built using 10-Fold cross validation, and RMSE was used as performance metric. The model with the top ranking (based on ranking over all datasets with sorted RMSE) was selected as the best algorithm for the retention prediction of small molecules in RPLC.

#### Algorithms

**Figure 6:**
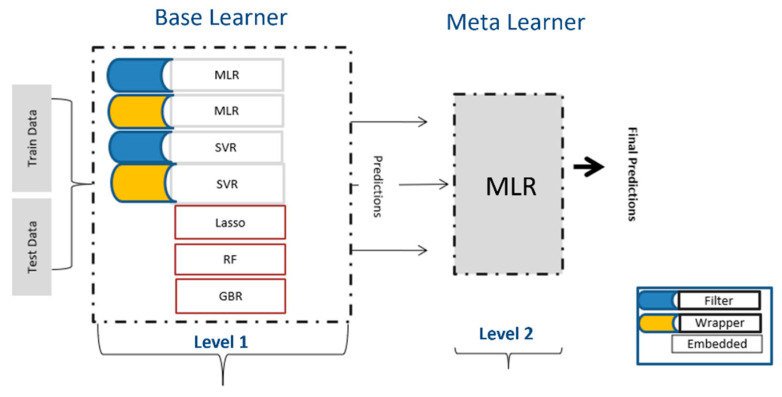
Architecture of Stacking used in this study.

As shown in Figure 6, five algorithms were used at level 1. LASSO regression is a type of linear regression that uses shrinkage by applying a penalty equal to the absolute value of the magnitude of coefficients (L1 regularization) [49]. The LASSO procedure encourages simple, sparse models (i.e., models with fewer parameters). This particular type of regression is well-suited for models showing high levels of multicollinearity. SVR with radial basis function kernel (RBF) was used to check the nonlinear dependencies. SVR provides the flexibility to define how much error is acceptable in the model and will find an appropriate line (or hyperplane in higher dimensions) to fit the data. The objective function of SVR is to minimize the coefficients, more specifically, the L2-norm of the coefficient vector [50]. The error term is handled in the constraints, where one set the absolute error less than or equal to a specified margin, called the maximum error, Є (epsilon).

RF and GBR both are ensemble learning methods and predict by combining the outputs from individual trees (tree-based regressions) [51,52]. They differ in the way the trees are built, that is, the order and the way the results are combined. The main objective of RF, which represents bagging, is to create several subsets of data from a training sample chosen randomly with replacement. Each collection of subset data is used to train their individual trees, resulting in an ensemble of different models. The average of all the predictions from different trees are used for predictions. In contrast to random forest regression, in GBR, the learners are learned sequentially with early learners, fitting simple models to the data and then analyzing data for errors. Consecutive trees (random sample) are fit at every step with the goal to improve the performance from the prior tree by applying different weights. Hence, in turn, this process converts weak learners into a better performing model.

### 3.6. Hyperparameter Optimization

To customize and obtain the most out of QSRR models, hyperparameters were configured using grid search that allowed models to be customized for specific task on all the datasets. Optimization was performed using 10-Fold cross validation, and RMSE was used as performance metric. Grid Search works by defining a search space or hyperparameter values in the form of a grid, and evaluates each and every position in that grid. The hyperparameters set with least RMSE were selected to build the prediction models. Grid search built in caret package itself was used for optimized parameter search.

### 3.7. Applicability Domain

The K-Nearest Neighbors (KNN) method has been used to calculate the AD of models. By this method, we calculate the distance of query compounds from a defined point within the descriptor space of the training data [38]. In this method, the average Euclidean distances of training molecules are calculated from their k-nearest training neighbors. An average distance value corresponding to a user-defined percentile is considered as a threshold. Those test compounds that have an average distance from their k-closest training neighbors greater than this threshold are reported to be out of the scope of the model’s applicability and vice versa. In the present study, a k = 5 number of nearest neighbors and a 95th percentile were selected to compute the AD.

### 3.8. Model Validation

Model validation step accounts for the fourth principle of OECD, and ensures the predictability and reliability of the QSRR model to evaluate the credibility of the model’s predictions on any new set of data. In the current study, the predictive abilities of QSRR regression models were assessed using 10-fold cross validation and external validation test data. In 10-fold cross validation, the compounds in the dataset were randomly divided into 10 partitions of equal size. Nine parts were used for training, while the last tenth was used as a test set. The process was repeated ten times in such a way that each sample was used exactly once as the test data in each cycle. There are many performance comparison metrices available in the literature to compare the generalization performance of fitted regression models, for example, (mean absolute error (mae), percentage mean absolute error (%mae), Root Mean Squared Error (rmse), percentage root mean square error (%rmse), and R^2^ for evaluating the predictive ability of quantitative structure-retention relationships (QSRR) models [53]; however, in the current study, Root Mean Squared Error (RMSE) and R^2^ are used for the same, the reason being that these two are considered an excellent general-purpose error metric for numerical predictions in most of the QSRR studies reported in the literature.

### 3.9. Tools and Software Used

RDKit library in Python version 5 September 2021 [54] and Chemicalize were used for calculation of molecular descriptor set. The statistical evaluation of the data, that is, preprocessing, feature selection, and regression prediction was performed using Caret package in R version 3.6 [55]. GGplot2 available in R was used for plotting observed versus prediction plots, and MS excel was used for plotting bar plots [56]. Applicability Domain toolbox, which is built in Matlab (R2019b), was used for the applicability domain calculation for prediction models [57]. Implementations and code used in this study can be accessed at: https://github.com/pkc533/QSRR_SMall-molecules, accessed on 1 January 2023.

## 4. Conclusions

Chromatographic separation of small molecules is a complex process and the development of new separation methods may be a long and costly process. QSRR proved to be an alternative solution enabling the selection of pre-optimal conditions based on in silico computations. However, such computational modeling approaches become tricky with an increasing number of chromatographic parameters. It is very challenging to use one type of algorithm over others since non-linear relationships between retention properties and the molecular descriptors may be present. The current study attempts to simplify the prediction modeling steps by taking a holistic approach that could be applied to any QSRR modeling for similar chemical compounds.

Since structures of compounds play a vital role in deciding separation patterns, the type of molecular descriptors and the way they have been calculated is crucial. The influence of change in pH on structure-derived molecular descriptors gave a deeper and better understanding of the molecules being studied and their retention pattern in the RPLC mode. The method of feature selection also affects the retention prediction performances. Stacking could be an excellent approach to combine predictions coming from different models, and could obtain better performances. QSRR modeling using a multitarget approach could be an advanced and more convenient way to deal with retention predictions with many experimental conditions.

We expect that the current study will provide the initial guiding points for a practical and effective method for analytical chemists working with LC platforms to obtain an optional working condition, as well as a way to improve the predictive confidence of studies.

## Figures and Tables

**Table 1:** Prediction performances of all models at pH 2.7.

Models	CV		External Test	
	RMSECV	R^2^	RMSE	R^2^
MLR_CFS	0.17	0.71	0.25	0.50
SVR_CFS	0.15	0.78	0.22	0.64
MLR_CFS	0.14	0.83	0.22	0.70
SVR_RFE	0.13	0.83	0.17	0.80
Lasso	0.13	0.84	0.20	0.70
RF	0.13	0.83	0.19	0.76
GBM	0.13	0.81	0.18	0.72
Stack	0.13	0.82	0.25	0.80

**Table 2:** Prediction performances of all models at pH 3.5.

Models	CV		External Test	
	RMSECV	R^2^	RMSE	R^2^
MLR_CFS	0.15	0.79	0.34	0.41
SVR_CFS	0.17	0.72	0.25	0.53
MLR_RFE	0.14	0.81	0.30	0.58
SVR_RFE	0.13	0.89	0.21	0.70
Lasso	0.13	0.82	0.22	0.66
RF	0.14	0.81	0.21	0.70
GBM	0.15	0.80	0.24	0.50
Stack	0.12	0.87	0.18	0.77

**Table 3:** Prediction performances of all models at pH 5.0.

Models	CV		External Test	
	RMSECV	R^2^	RMSE	R^2^
MLR_CFS	0.15	0.81	0.41	0.42
SVR_CFS	0.19	0.78	0.26	0.63
MLR_RFE	0.15	0.82	0.26	0.64
SVR_RFE	0.14	0.85	0.19	0.83
Lasso	0.13	0.87	0.23	0.71
RF	0.14	0.87	0.22	0.75
GBM	0.14	0.85	0.23	0.69
Stack	0.12	0.87	0.21	0.75

**Table 4:** Prediction performances of all models at pH 6.5.

Models	CV		External Test	
	RMSECV	R^2^	RMSE	R^2^
MLR_CFS	0.20	0.76	0.31	0.58
SVR_CFS	0.23	0.73	0.35	0.44
MLR_RFE	0.16	0.87	0.29	0.63
SVR_RFE	0.16	0.88	0.19	0.84
Lasso	0.16	0.81	0.28	0.71
RF	0.15	0.87	0.20	0.84
GBM	0.15	0.88	0.15	0.90
Stack	0.13	0.90	0.18	0.85

**Table 5:** Prediction performances of all models at pH 8.0.

Models	CV		External Test	
	RMSECV	R^2^	RMSE	R^2^
MLR_CFS	0.21	0.77	0.26	0.71
SVR_CFS	0.22	0.76	0.29	0.64
MLR_RFE	0.21	0.83	0.22	0.79
SVR_RFE	0.17	0.87	0.15	0.91
Lasso	0.15	0.89	0.30	0.70
RF	0.15	0.86	0.17	0.88
GBM	0.16	0.86	0.15	0.89
Stack	0.14	0.92	0.12	0.93

**Table 6:** Applicability domain calculated for each compound in test set. (Errors in columns 2,3,4,5,6 are the errors of prediction from all the models specific for compounds. Distances in columns 5,6,7,8,9 are their distances calculated using KNN fixed methods). Errors are based on back-transformed retention times (min unit).

Compound	Error pH 2.7	Error pH 3.5	Error pH 5.0	Error pH 6.5	Error pH 8.0	Distance pH 2.7	Distance pH 3.5	Distance pH 5.0	Distance pH 6.5	Distance pH 8.0	Applicability
23dideoxyadenosine	0.49	1.67	0.47	1.49	2.82	13.86	13.57	12.87	13.15	13.35	In
mefenamic acid	8.07	0.00	4.30	5.44	1.60	11.32	11.34	11.43	11.37	11.40	In
cytosine	5.11	1.37	2.62	1.81	1.65	9.51	9.16	8.81	9.06	9.26	In
gallic acid	2.76	2.09	0.11	0.21	0.20	8.39	8.45	8.44	8.48	8.53	In
4aminosalicylic acid	0.19	3.37	0.80	0.05	0.37	5.84	6.20	6.21	6.14	6.21	In
2deoxyguanosine	1.42	2.08	1.91	2.55	0.47	12.77	12.39	12.37	12.64	12.36	In
miconazole	3.82	9.03	34.90	23.87	6.21	21.72	21.81	21.89	21.47	21.52	Out
chlordiazepoxide	0.32	4.50	3.49	0.00	0.84	11.50	11.54	11.70	11.52	11.86	In
4nitrophenol	1.96	4.98	4.98	4.41	1.96	7.66	7.73	7.77	8.05	9.12	In
coumarin	3.26	1.95	0.31	0.94	1.65	7.44	7.49	8.03	8.27	8.50	In
Threshold						15.87	15.86	15.88	15.64	14.99	

## Data Availability

All data are contained within the article and supplemental information.

## Supplementary Materials

The following supporting information can be downloaded at: https://www.mdpi.com/article/10.3390/molecules28041696/s1.

## Abbreviations

MLR_cfs	Multiple Linear regression on correlation based features selected
SVM_cfs	Support Vector Machine on correlation based features selected
GBM	Gradient Boosting Model
RF	Random Forest: tR- Retention Time
CFS	Correlation based feature Selection
RFE	Recursive Feature Elimination
AD	Applicability Domain
RMSE	Root mean Squared Error
CV	Cross Validation
R^2^	Coefficient of Correlation
LC	Liquid Chromatography
