# Supplementary material for: Quantitative Structure Retention-Relationship Modeling: Towards an Innovative General-Purpose Strategy
Source: Molecules. 2023 Feb 10;28(4):1696. doi: 10.3390/molecules28041696 (PMC9964055; doi:10.3390/molecules28041696)
Supplement: Supplementary file 1 [file molecules-28-01696-s001.zip › molecules-2173883-SI.pdf]

Supplementary Information

Title: Quantitative structure retention-relationship modelling: Towards an innovative general-purpose strategy

Authors: Priyanka Kumari[a,b*], Thomas Van Laethem[a,b], Philippe Hubert[a], Marianne Fillet[b], Pierre-Yves Sacré[a], Cédric Hubert[a*]

a. University of Liège (ULiege), CIRM, Laboratory of Pharmaceutical Analytical Chemistry, Liège, Belgium

b. University of Liège (ULiege), CIRM, Laboratory for the Analysis of Medicines, Liège, Belgium

---

S1: Illustration of molecular descriptor calculation with an example

There were two tools used for molecular descriptor calculation: [1] RdKit [2] Chemicalize

- Values of LogD was calculated using Chemicalize at each pH

Method of calculation of other descriptors:

- Step1: Smile strings of compounds into Chemicalize
- Step2: Retrieve Smile strings of all microspecies and their distributions at all pH
- Step3: Calculate molecular descriptors of every microspecies from RdKit
- Step4: Calculate weighted average of molecular descriptors at each pH using formula below

$$FV_{ph} = \frac{\sum_{i=1}^{n} MS_i * D_i}{\sum_{i=1}^{n} D_i}$$

Where , FV_{ph} = Weighted average, MS_i = Descriptor value for microspecies and,

D_i = %Distribution of microspecies, n = no. of microspecies , ph = Specific pH at which final value is being calculated

Step-1

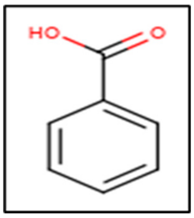

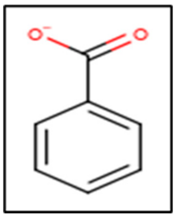

[b]Benzoic Acid (MS1) - C1=CC=C(C=C1)C(=O)O [b]Benzoate(MS2)- MS1C1=CC=C(C=C1)C(=O)[O-]

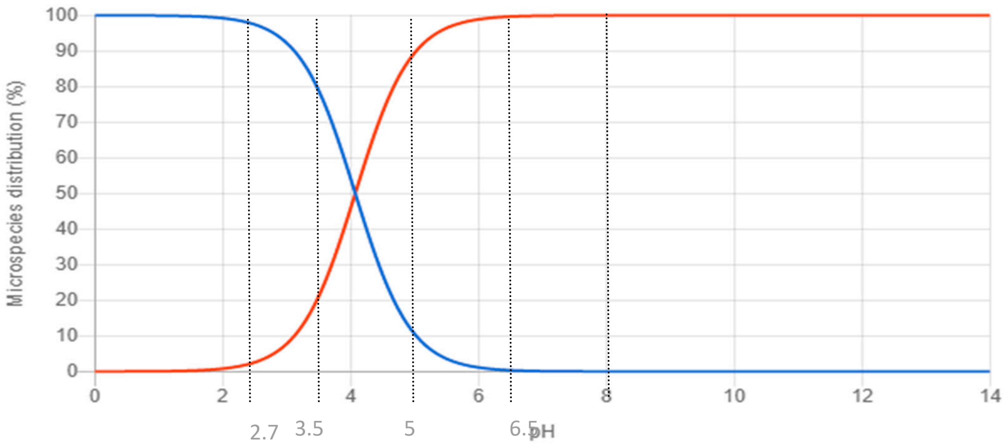

Step2: Microspecies distribution calculation using Chemicalize

Condition	MS1	MS2
pH2.50	0.97	0.03
pH3.50	0.79	0.21
pH5.00	0.11	0.89
pH6.50	0.00	1.00
pH8	0.00	1.00

Step3: Calculate molecular descriptors of every microspecies from RdKit

Microspecies	PEOE_VSA7
MS1	12.13
Ms2	5.56

Step4: Calculate weighted average of molecular descriptors at each pH :

Final value of PEOE_VSA7

[1] At pH2.7

= (12.13*0.97 + 5.56*0.03)/1

= 11.76 + 0.166

= 11.92

[2] At pH 3.5

= (12.13*0.79 + 5.56*0.21)/1

= 9.58 + 1.16

= 10.74

[3] At pH 5

= (12.13*0.11 + 5.56*0.89)/1

= 1.33 + 4.94

= 6.27

[4] At pH 6.5

= (12.13*0.0 + 5.56* 1.0)/1

=5.56

[5] At pH 8

= (12.13*0.0 + 5.56* 1.0)/1

= 5.56

Final Value of molecular descriptors: -

**Condition**	**Final Value**<br>**PEOE_VSA7**

pH 2.7	11.92
pH 3.5	10.74
pH5	6.27
pH6.5	5.56
pH 8	5.56

Table S2: Name of features used to start QSRR modeling

MolWt	EState_VSA2	fr_ArN	fr_quatN	MinEStateIndex	PEOE_VSA10	SlogP_VSA3	VSA_EState6
logD	EState_VSA3	fr_aryl_methyl	FractionCSP3	MinPartialCharge	PEOE_VSA11	SlogP_VSA4	VSA_EState7
Asymmetric.atom.count	EState_VSA4	fr_benzene	FSP3	Molar.refractivity	PEOE_VSA12	SlogP_VSA5	VSA_EState8
Atom.count	EState_VSA5	fr_bicyclic	HallKierAlpha	MolLogP	PEOE_VSA13	SlogP_VSA6	VSA_EState9
BalabanJ	EState_VSA6	fr_C_O	Heavy.atom.count	MolMR	PEOE_VSA14	SlogP_VSA7	
BertzCT	EState_VSA7	fr_C_O_noCOO	HeavyAtomMolWt	NHOHCount	PEOE_VSA2	SlogP_VSA8	
Chi0	EState_VSA8	fr_COO	Hetero.ring.count	NOCount	PEOE_VSA3	SMR_VSA1	
Chi0n	EState_VSA9	fr_COO2	Hydrogen.bond.acceptor.count	NumAliphaticHeterocycles	PEOE_VSA6	SMR_VSA10	
Chi0v	FpDensityMorgan1	fr_ether	Hydrogen.bond.donor.count	NumAliphaticRings	PEOE_VSA7	SMR_VSA3	

Chi1	FpDensityMorgan2	fr_halogen	Ipc	NumAromaticCarbocycles	PEOE_VSA8	SMR_VSA5	
Chi1n	FpDensityMorgan3	fr_imidazole	Kappa1	NumAromaticHeterocycles	PEOE_VSA9	SMR_VSA6	
Chi1v	fr_Al_COO	fr_Ndealkylation1	Kappa2	NumAromaticRings	Polarizability	SMR_VSA7	
Chi2n	fr_Al_OH	fr_NH0	Kappa3	NumHAcceptors	qed	SMR_VSA9	
Chi2v	fr_Al_OH_noTert	fr_NH1	LabuteASA	NumHDonors	Ring.count	TPSA	
Chi3n	fr_amide	fr_NH2	MaxAbsEStateIndex	NumHeteroatoms	Rotatable.bond.count	VSA_EState1	
Chi3v	fr_aniline	fr_Nhpyrrole	MaxAbsPartialCharge	NumRotatableBonds	SlogP_VSA1	VSA_EState10	
Chi4n	fr_Ar_COO	fr_para_hydroxylation	MaxEStateIndex	NumSaturatedHeterocycles	SlogP_VSA10	VSA_EState2	
Chi4v	fr_Ar_N	fr_phenol	MaxPartialCharge	NumSaturatedRings	SlogP_VSA11	VSA_EState3	
EState_VSA1	fr_Ar_NH	fr_phenol_noOrthoHbond	MinAbsEStateIndex	NumValenceElectrons	SlogP_VSA12	VSA_EState4	
EState_VSA10	fr_Ar_OH	fr_pyridine	MinAbsPartialCharge	PEOE_VSA1	SlogP_VSA2	VSA_EState5	

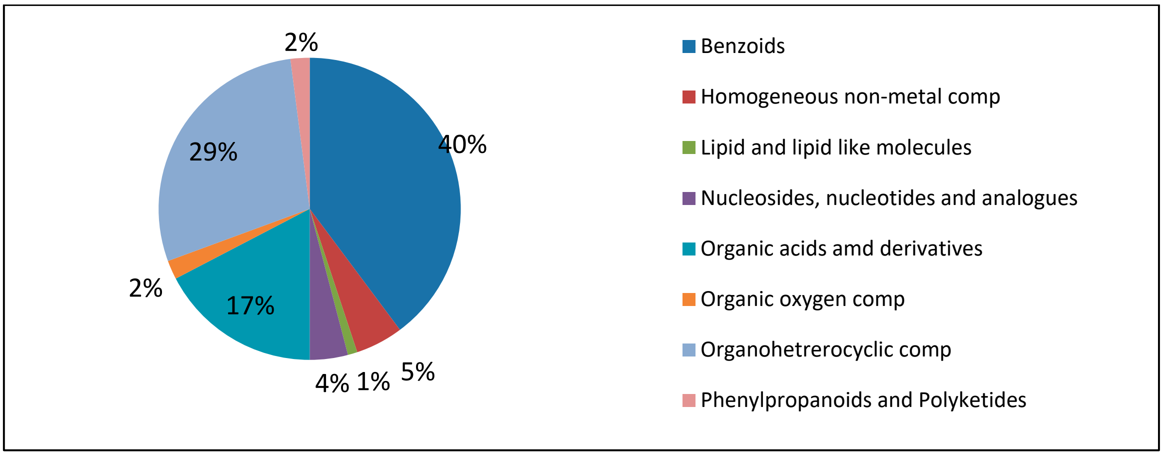

Figure S3:Chemical taxonomy of the molecules in the dataset

Table S4: Important features selected by each algorithm on data at each pH

pH	Total	Descriptors
2.7	5	MolLogP, logD , NHOHCount , fr_Ar_NH, PEOE_VSA6
3.5	3	MolLogP, logD, NHOHCount
5	4	Polarizability, MolLogP, logD, PEOE_VSA6
6.5	3	MolLogP,logD,PEOE_VSA6
8	3	MolLogP , logD, PEOE_VSA6

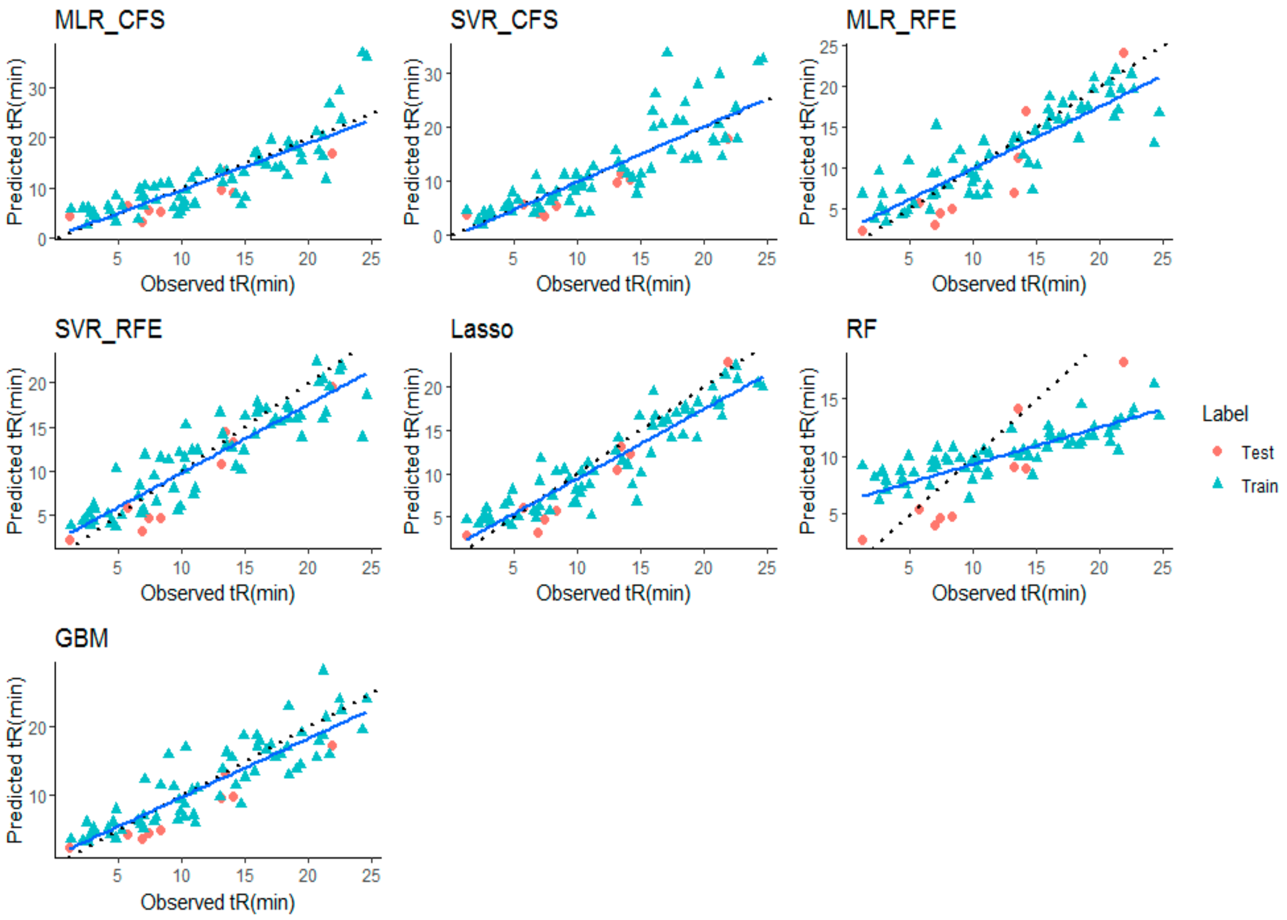

S5: Predicted Vs. Experimental Retention times (in min.) for all models at pH2.7 (Blue line- fit , Black dashed line- identity line)

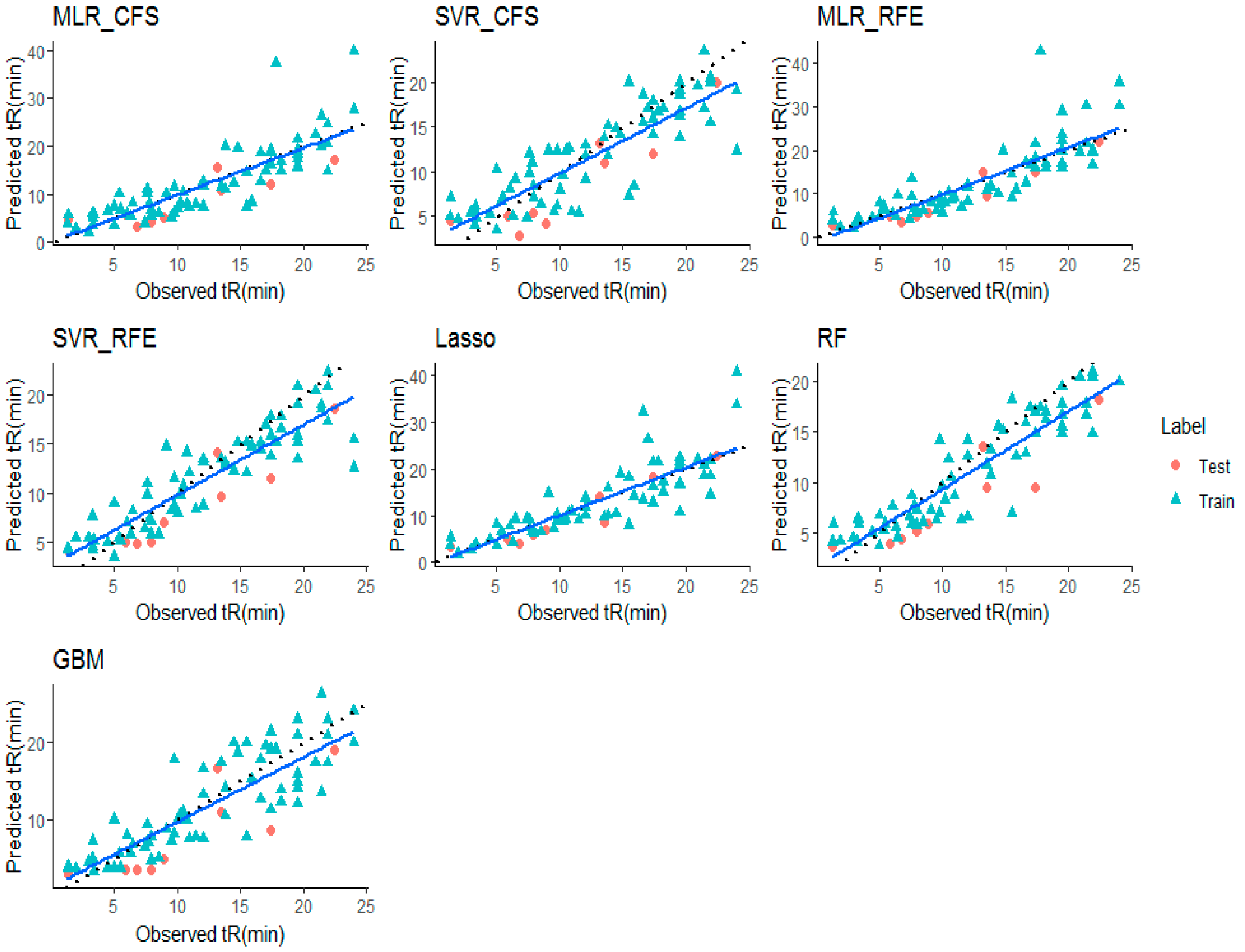

S6: Predicted Vs. Experimental Retention times (in min.) for qsrr models at pH 3.5((Blue line- fit , Black dashed line- identity line)

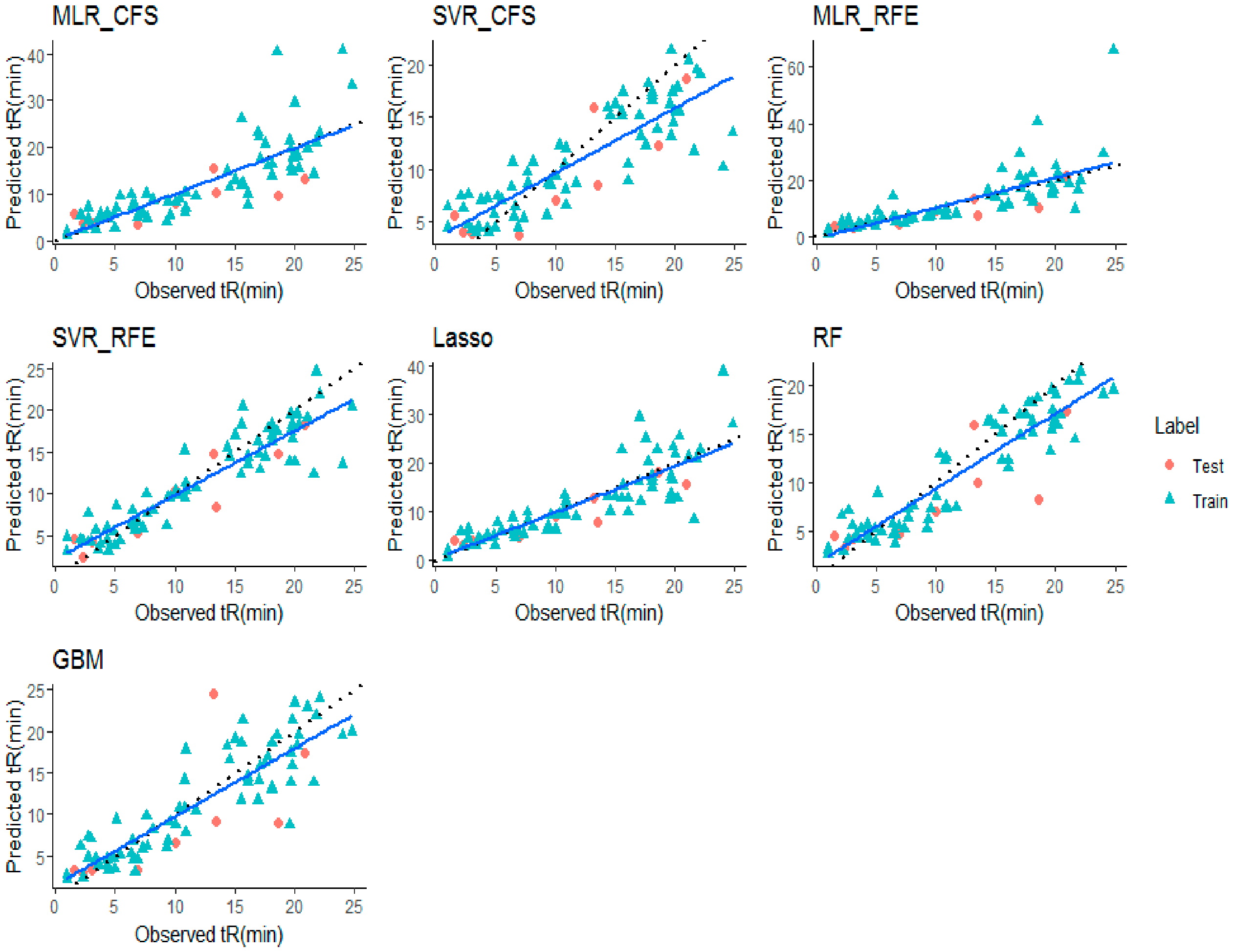

S7: Predicted Vs. Experimental Retention times (in min.) for qsrr models at pH 5 (Blue line- fit , Black dashed line- identity line)

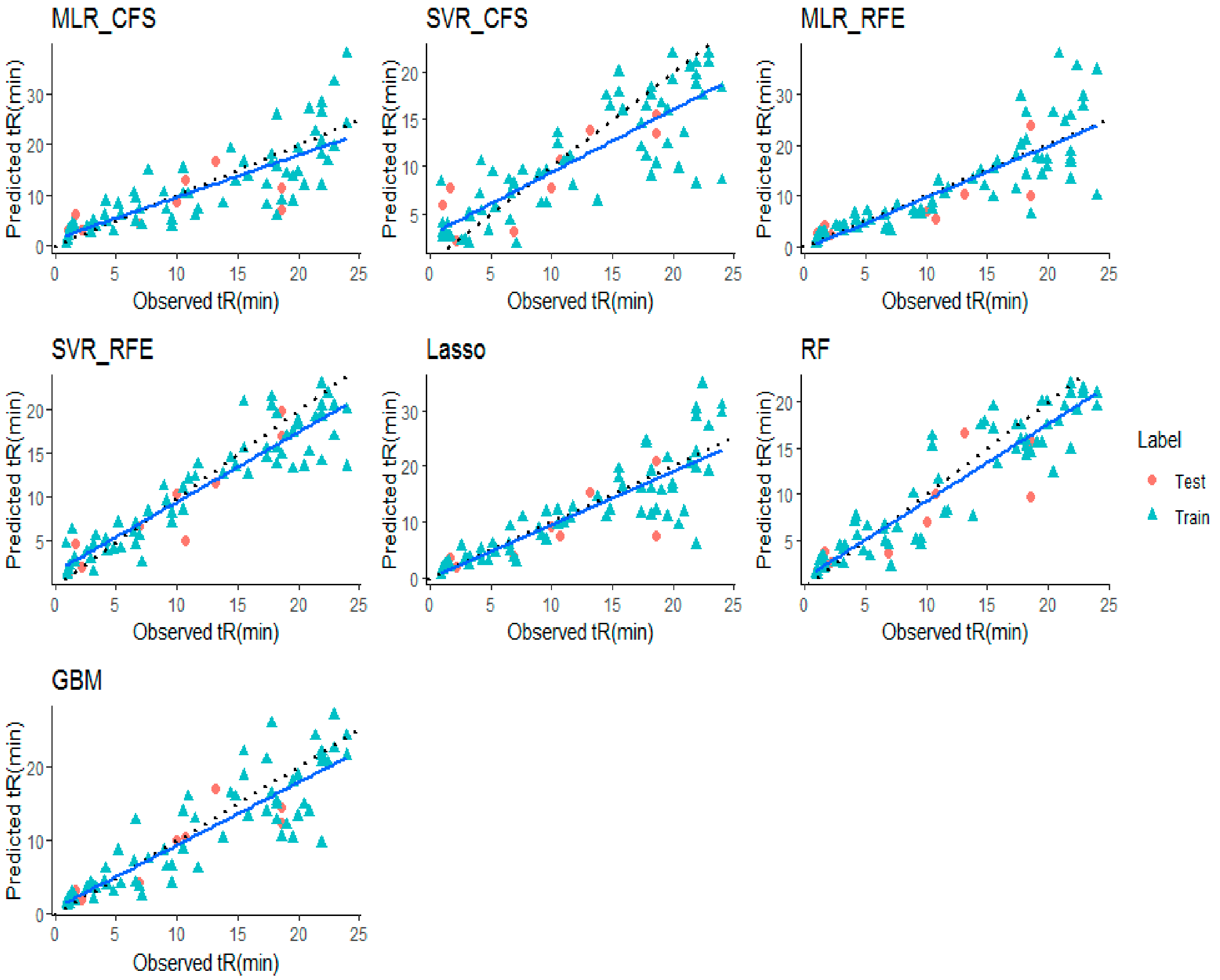

S8: Predicted Vs. Experimental Retention times for qsrr models at pH 6.5 (Blue line- fit, Black dashed line- identity line)

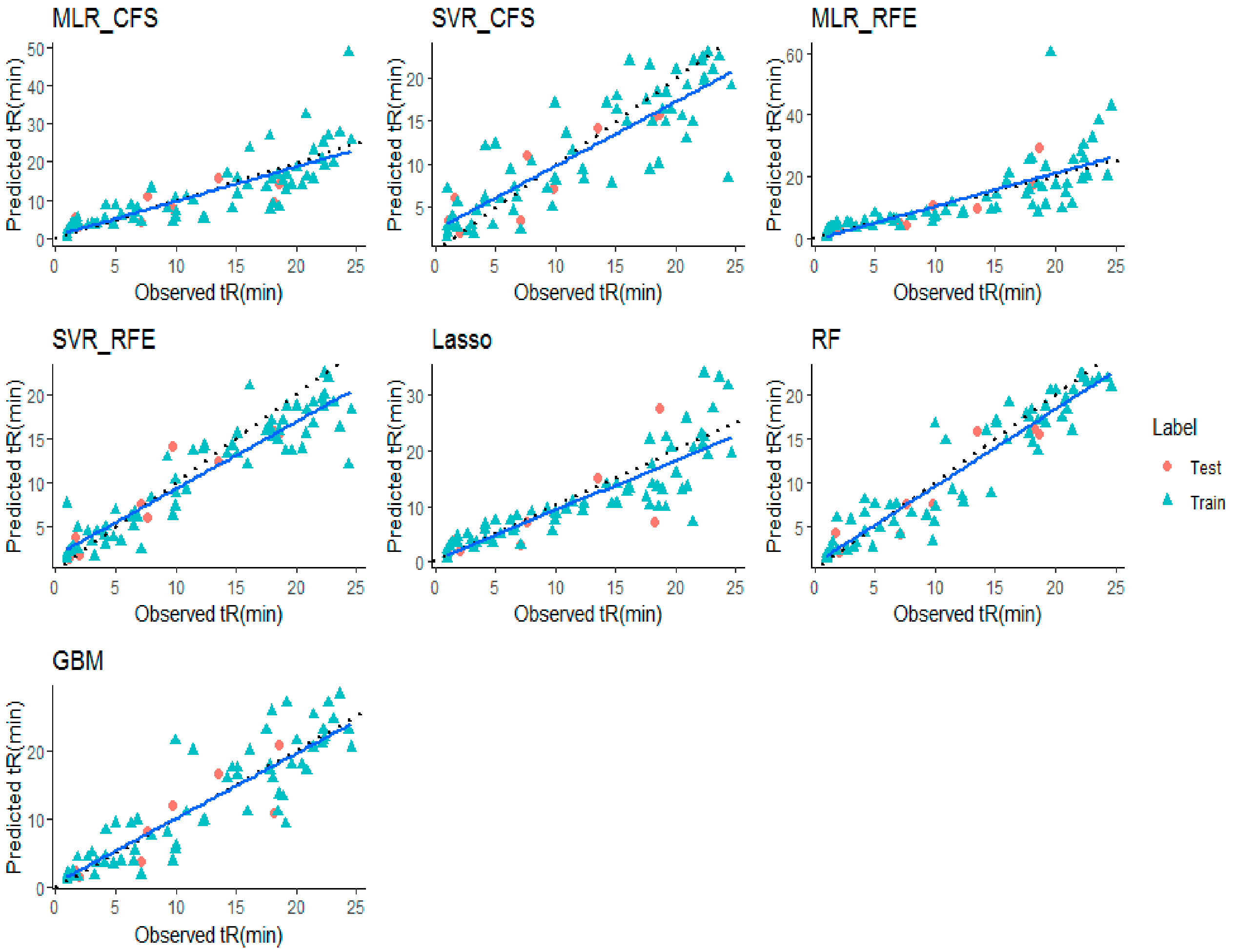

S9: Predicted Vs. Experimental Retention times for qsrr models at pH 8 (Blue line- fit, Black dashed line- identity line)

S10: Parameters used by prediction models at all pH

Models at pH2.7:

SVR_CFS	Sigma = 0.362	C = 1
SVR_RFE	Sigma = 0.048	C=1
Lasso	Aplha = 1	Lambda = 0.014
RF	Mtry = 73	
GBM	n. trees = 150,<br>Shrinkage = 0.1	Interaction depth =2,<br>n. minobsinnode = 10

Models at pH3.5:

SVR_CFS	Sigma = 0.122	C = 1
SVR_RFE	Sigma = 0.075	C=1
Lasso	Aplha = 0.1	Lambda = 0.048
RF	Mtry = 73	
GBM	n. trees = 150,<br>Shrinkage = 0.1	Interaction depth =2,<br>n.minobsinnode = 10

Models at pH 5:

SVR_CFS	Sigma = 0.353	C = 1

SVR_RFE	Sigma = 0.054	C=1
Lasso	Aplha = 0.1	Lambda = 0.058
RF	Mtry = 73	
GBM	n.trees = 150,<br>Shrinkage = 0.1	Interaction depth =2,<br>n.minobsinnode = 10

Models at pH 6.5:

SVR_CFS	Sigma = 0.313	C = 1
SVR_RFE	Sigma = 0.042	C=1
Lasso	Aplha = 0.1	Lambda = 0.02
RF	Mtry = 147	
GBM	n.trees = 150,<br>Shrinkage = 0.1	Interaction depth =3,<br>n.minobsinnode = 10

Models at pH 8:

SVR_CFS	Sigma = 0.319	C = 1
SVR_RFE	Sigma = 0.110	C=1
Lasso	Aplha = 0.1	Lambda = 0.024
RF	Mtry = 147	
GBM	n.trees = 150,<br>Shrinkage = 0.1	Interaction depth =2,<br>n.minobsinnode = 10

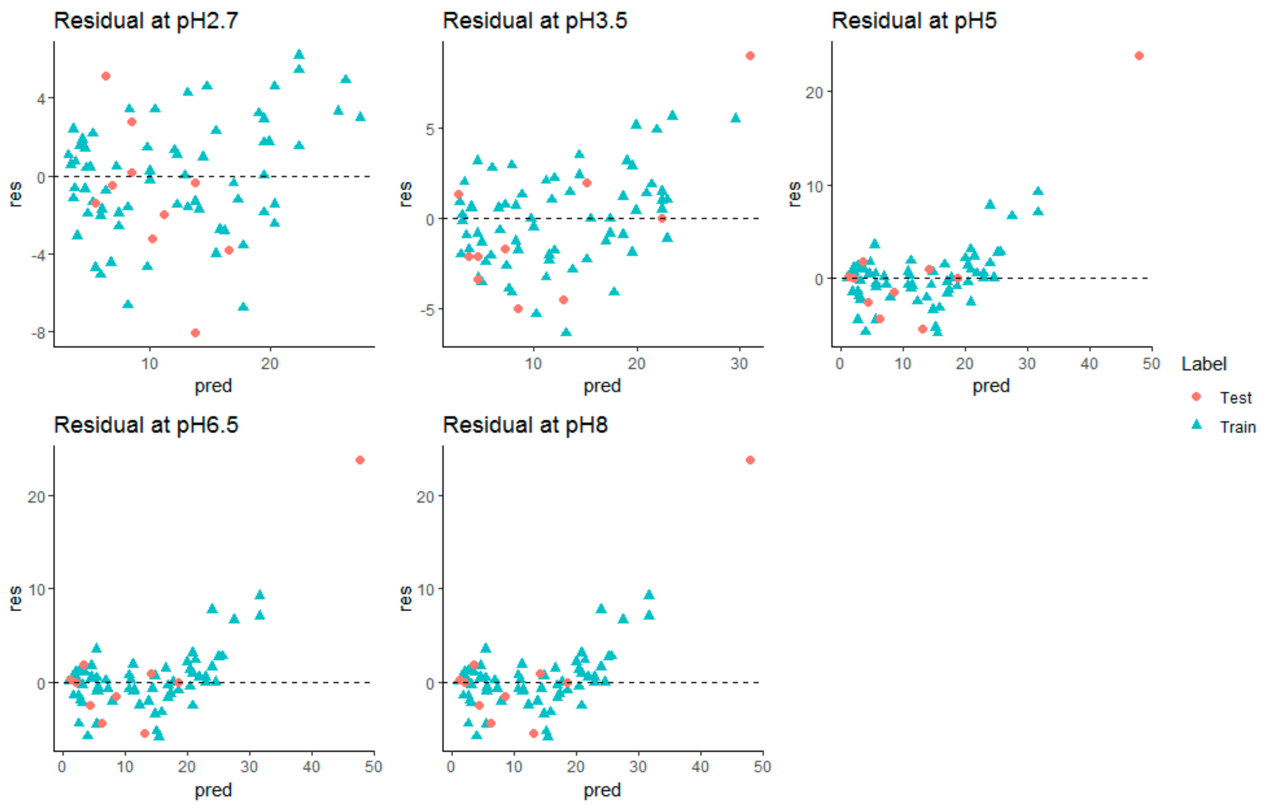

S11: Residual plots (in min) for Stacking model for all dataset(with Miconazole)
